# Transmembrane Protein ANTXR1 Regulates *γ*-Globin Expression by Targeting the Wnt/*β*-Catenin Signaling Pathway

**DOI:** 10.1155/2022/8440422

**Published:** 2022-07-30

**Authors:** Tingting Jin, Zhaojun Zhang, Yuanyuan Han, Di Li, Juan Liu, Minmin Jiang, Junwei Zhu, Ryo Kurita, Yukio Nakamura, Fangfang Hu, Yongjie Xu, Xiangdong Fang, Shengwen Huang, Zhaolin Sun

**Affiliations:** ^1^School of Medicine, Guizhou University, Guiyang, Guizhou 550025, China; ^2^Prenatal Diagnosis Center, Guizhou Provincial People's Hospital, Guiyang, Guizhou 550002, China; ^3^CAS Key Laboratory of Genome Sciences and Information, Beijing Institute of Genomics, Chinese Academy of Sciences, Beijing 100101, China; ^4^Cell Engineering Division, RIKEN BioResource Center, Tsukuba, Ibaraki 305-0074, Japan; ^5^Department of Laboratory, Guizhou Provincial People's Hospital, Guiyang, Guizhou 550002, China; ^6^NHC Key Laboratory of Pulmonary Immunological Diseases, Guizhou Provincial People's Hospital, Guiyang, Guizhou 550002, China; ^7^Department of Urology, Guizhou Provincial People's Hospital, Guiyang, Guizhou 550002, China

## Abstract

Reactivation of fetal hemoglobin (HbF, *α*2*γ*2) alleviates clinical symptoms in patients with *β*-thalassemia and sickle cell disease, although the regulatory mechanisms of *γ*-globin expression have not yet been fully elucidated. Recent studies found that interfering with the expression of the membrane protein ANTXR1 gene upregulated *γ*-globin levels. However, the exact mechanism by which ANTXR1 regulates *γ*-globin levels remains unclear. Our study showed that overexpression and knockdown of ANTXR1 in K562, cord blood CD34^+^, and HUDEP-2 cells decreased and increased *γ*-globin expression, respectively. ANTXR1 regulates the reactivation of fetal hemoglobin (HbF, *α*2*γ*2) in K562, cord blood CD34^+^, and adult peripheral blood CD34^+^ cells through interaction with LRP6 to promote the nuclear entry of *β*-catenin and activate the Wnt/*β*-catenin signaling pathway. The overexpression or knockdown of ANTXR1 on *γ*-globin and Wnt/*β*-catenin signaling in K562 cells was reversed by the inhibitor XAV939 and the activator LiCl, respectively, where XAV939 inhibits the transcription of *β*-catenin in the Wnt pathway, but LiCl inhibits GSK3-*β*. We also showed that the binding ability of the rank4 site in the transcriptional regulatory region of the SOX6 gene to c-Jun was significantly increased after overexpression of ANTXR1 in K562 cells. SOX6 protein expression was increased significantly after overexpression of the c-Jun gene, indicating that the transcription factor c-Jun initiated the transcription of SOX6, thereby silencing *γ*-globin. Our findings may provide a new intervention target for the treatment of *β*-hemoglobinopathies.

## 1. Introduction

*β*-Hemoglobinopathies are common autosomal recessive hematological disorders caused by mutations in the *β*-globin gene, of which *β*-thalassemia and sickle cell disease (SCD) are the two most important conditions. Reactivation of fetal hemoglobin (HbF, *α*_2_*γ*_2_) ameliorates the clinical symptoms of *β*-thalassemia and sickle cell disease (SCD) [1–3]. Transcription factors, such as BCL11A, SOX6, KLF1, and ZBTB7A, play critical roles in the expression of *γ*-globin genes (*HBG2* and *HBG1*), and their molecular mechanisms have only been partially elucidated [3–8]. Since *γ*-globin gene expression has an intricate interplay of many factors at different levels, the molecular mechanism is not yet fully understood.

Anthrax toxin receptor 1 (ANTXR1), a type I transmembrane protein, also known as tumor endothelial marker 8 (TEM8), was initially identified because of its overexpression in the endothelial cells (ECs) that is associated with human colorectal cancer [9–12] Previous studies had not shown an association between ANTXR1 and hematological phenotypes until the recent finding that a single nucleotide polymorphism (SNP, rs4527238, T>C) in intron 9 of the *ANTXR1* gene was associated with the regulation of HbF levels in Saudi patients with SCD [13]. Several other studies and our research reconfirmed that the genotypes of two SNPs (rs4527238 and rs35685045) in the *ANTXR1* gene were associated with HbF levels in individuals with *β*-thalassemia minor and sickle cell anemia [14]. These findings suggest that ANTXR1 is involved in the regulation of *γ*-globin gene expression. All recent studies indicate that genetic mutations of ANTXR1 are associated with HbF expression in *β*-thalassemia and SCD; ANTXR1 gene may be a major HbF modulator leading to potential therapeutic options.

Since ANTXR1 is a membrane protein, we speculate that ANTXR1 may regulate the expression of *γ*-globin genes through translocation and cell signaling. Earlier studies have found that the direct interaction of ANTXR1 with LDL-receptor-related protein (LRP) is an essential process for mammalian cell death induced by anthrax toxin [15]. Subsequently, using the protein-protein interaction database (STRING 9.0), we found that the protein, LRP6, was the most likely to interact with ANTXR1 [13] and affected the activity of the Wnt/*β*-catenin signaling pathway in some tissues and tumor cells [16, 17]. However, the correlations between this pathway and *γ*-globin regulation have not yet been established.

In the present study, we overexpressed and interfered with the *ANTXR1* gene in different erythroid differentiated cell models and analyzed the expression and interaction of related proteins in the Wnt/*β*-catenin signaling pathway. The study sought to investigate the involvement of Wnt/*β*-catenin signaling in ANTXR-mediated *γ*-globin expression.

## 2. Materials and Methods

### 2.1. Cell Culture

K562 cells were cultured in RPMI-1640 medium supplemented with 10% fetal bovine serum (FBS) and 1% streptomycin/penicillin at 37°C in 5% carbon dioxide. Umbilical cord blood (CB) and adult peripheral blood (AB) samples were obtained from the Obstetrics Department of Beijing Maternity Hospital with informed consent for inclusion in the study signed by the donors. The mononuclear cells from CB and AB were isolated using lymphocyte separation solution Ficoll-Paque™ Plus (GE Healthcare, #17144002); then, primary CB and AB CD34^+^ cells were purified using the EasySep™ Human CD34 Positive Selection Kit II (STEMCELL, #17896, #17856) according to the manufacturer's instructions. Isolated CD34^+^ cells were cultured in StemSpan™ SFEM II medium for two phases [13]. Phase 1 involved cell proliferation and expansion for 7 days, and phase 2 involved erythroid differentiation for 16 days. The HUDEP-2 cell line donated by RIKEN Tsukuba Branch, Ibaraki, Japan, was cultured and differentiated as previously described [18].

### 2.2. Overexpression and RNA Interference of ANTXR1

The mRNA sequence of human *ANTXR1* gene transcript (NM032208.2) from K562 cells was used to clone cDNA using RT-PCR. Then, it was inserted into the cloning vector pHAGE-fEF-1a-IRES-ZsGreen-2 (Beijing Institute of Genomics, Chinese Academy of Sciences). Five short hairpin (sh) RNAs targeting the *ANTXR1* gene (ANTXR1-shRNA1-5) and one negative control shRNA (NC-shRNA) were designed and synthesized. The sequences of the shRNAs are listed in Supplemental Table 1. The NC-shRNA and ANTXR1-shRNA sequences were designed by online software (https://www.sigmaaldrich.cn/cn/zh/semi-configurators/shrna?activelink=productsearch). The synthesized sequences of ANTXR1-shRNA1-5 and NC-shRNA were inserted into the empty vector pSIH1-H1-copGFPshRNA vector. The constructed *ANTXR1* overexpression and interference plasmids were cotransfected with the packaging plasmid into 293T cells. Green fluorescent protein expression was observed under an inverted fluorescence microscope after 48 hours of transfection. Successfully packaged overexpressed and interfered *ANTXR1* lentiviruses were used to infect CD34^+^ cells and HUDEP-2 cells on day 2 of erythroid differentiation and K562 cells, respectively.

### 2.3. RT-qPCR

Total RNA was extracted using the TRIZOL kit (Life Technologies, #15596018) and was reverse transcribed into cDNA using the PrimeScript RT reagent Kit with gDNA Eraser (TaKaRa, #RR047A). The gene expression of the *ANTXR1*, *γ-globin*, *LRP6*, *CTNNB1* (*β*-catenin gene), *c-Jun*, *CCND1* (cyclin D1gene), and *β*-globin was detected by RT-PCR, and their relative expression was calculated by the 2^-*ΔΔ*Ct^ method. The primers are listed in Supplemental Table 2.

### 2.4. Flow Cytometry

A total of 1 × 10^6^ CD34^+^ cells were collected and washed twice with 1 × PBS. The cells were permeabilized with Triton X-100 (Beyotime, #P0096) for 20 minutes, and washed twice with 1 × PBS after fixation in 4% paraformaldehyde solution for 15 minutes. Then, immunostaining was carried out followed by the addition of PE-labeled HbF monoclonal antibody (Becton, Dickinson and Company, #560041). The cells were incubated for 30 minutes at room temperature in the dark, followed by a wash with 1 × PBS. The cells were then analyzed by a fluorescence-activated cell sorting (FACS) machine using an isotype control to exclude nonspecific binding.

### 2.5. Western Blot

Total protein was extracted from 1 × 10^6^ cells, and the protein concentration was determined by the BCA assay. 20 *μ*g of protein sample solution was used for western blot analysis; the primary antibodies, anti-ANTXR1 (ab21270), anti-fetal hemoglobin (ab137096), anti-hemoglobin subunit beta antibody (ab214049), anti-LRP6 (ab75358), anti-cyclin D1 (ab40754), anti-GAPDH (ab181602), anti-GFP (ab290), anti-Flag antibody (ab205606) from Abcam, and anti-*β*-catenin (#8480) and anti c-Jun (#9165s) were purchased from Cell Signaling Technology. The membrane was then washed three times with TBST, the secondary HRP-conjugated antibody was applied, and the membrane was incubated for 1 hour at room temperature. Finally, immunoreactive protein bands were visualized using the ECL system, and the optical density of the protein bands was quantified using ImageJ software (http://imagej.nih.gov/ij).

### 2.6. Immunofluorescence Staining

A total of 1 × 10^6^ K562 cells were collected and centrifuged to remove the supernatant, washed twice with 1 × PBS, resuspended in 20 *μ*L PBS, then dropped onto a glass slide to air-dry. Following fixation in 4% paraformaldehyde for 30 minutes, the slides were permeabilized for 15 minutes in Triton X-100 (Beyotime, #P0096) at room temperature and then rinsed three times with 1 × PBS. Blocking was performed with 5% BSA (blocking solution) for 30 minutes at room temperature. Primary anti-*β*-catenin (Cell Signaling Technology, #84803) was diluted at 1 : 500 in 5% BSA and followed by incubation overnight at 4°C. After three washes in PBS, the rabbit IgG (H+L) highly crossadsorbed secondary antibody (Thermo Fisher Scientific, #A-11037) was added and incubated for 1 hour in the dark, followed by three rinses with PBS. Finally, the samples on slides mounted with 5% glycerol were observed with a confocal laser scanning microscope to locate *β*-catenin.

### 2.7. TOP-/FOP-Flash Luciferase Reporter Assay

293T cells were plated into 48-well plates at a concentration of 1 × 10^5^ cells per well. The cells were then cotransfected with 100 ng of either TOP flash (T-cell factor reporter plasmid) or FOP flash (mutant T-cell factor reporter plasmid) expression plasmids (Millipore), 150 ng of *ANTXR1* overexpression plasmid vector, *ANTXR1* knockdown plasmid vector or control vector, and 10 ng of internal control pRL-TK. After 36 hours, the cells were collected to detect luciferase activity according to the instructions of the Dual-Luciferase Reporter Assay System (Promega, E1910). Relative *β*-catenin activation was determined by the TOP-FLASH/FOP-FLASH ratio.

### 2.8. Separation of Nuclear and Cytoplasmic Proteins

To detect the protein levels of *β*-catenin in the nucleus and cytoplasm separately, cytoplasmic proteins and nuclear proteins were extracted using the Nuclear Protein and Cytoplasmic Protein Extraction Kit (Beyotime, #P0027) at present of protease inhibitor (Beyotime, #ST505). The samples were vortexed vigorously for 15 seconds and kept in an ice bath for 15 minutes. After the addition of cytoplasmic protein extraction buffer, the lysate was vortexed and centrifuged at 16000 × g for 10 minutes at 4°C, and the supernatant (cytoplasmic protein) then transferred to a clean prechilled tube. The 2nd part of the precipitate was solubilized with nucleoprotein extraction buffer, vortexed vigorously for 15 seconds, and incubated on ice for 30 minutes. The supernatant (nuclear protein) was collected by centrifugation at 16,000 × g for 10 minutes at 4°C. The protein *β*-catenin in the cytoplasm and nucleus was determined by western blotting.

### 2.9. Coimmunoprecipitation (CoIP) Analysis

The coimmunoprecipitation (Co-IP) assay was used to examine the interaction between the proteins ANTXR1 and LRP6 in K562 cells. The ANTXR1–GFP and LRP6-Flag fusion plasmids were verified through DNA sequencing and transfected in pairs into the K562 cells. Then, the K562 cells were collected and washed twice with PBS, followed by the addition of ice-cold IP lysis/wash buffer (2X 50 mL, 0.025 M Tris, 0.15 M NaCl, 0.001 M EDTA, 1% NP-40, and 5% glycerol; pH 7.4). Lysates were centrifuged at 14,000 × g and 4°C for 15 min. The supernatant was incubated overnight at 4°C with the anti-GFP antibody (ab290, Abcam). Subsequently, 50 *μ*L agarose was added, and the mixture was incubated for 1 h at 4°C with constant rotation. The beads were washed extensively and boiled in SDS loading buffer. The expression of ANTXR1 and LRP6 was determined through western blotting.

### 2.10. ChIP-qPCR

A total of 1 × 10^7^ K562 cells were collected. 1 mL of 9% formaldehyde solution was added to the cells and left at room temperature for 10 minutes, and then, 1.57 mL of 1 M glycine solution was added and left at room temperature for 5 minutes. The mixture was centrifuged at 300 × g for 5 minutes at 4°C. After sonication of the pellet, the soluble chromatin was centrifuged at 14,000 rpm for 15 minutes at 4°C to separate the supernatant. 20 *μ*L of the supernatant was collected as an input sample (positive control). 500 *μ*L of supernatant was added to the negative control IgG antibody (Abcam, ab172730) and c-Jun antibody (Abcam, ab31419), followed by incubation overnight at 4°C. The immunoprecipitated complexes were eluted with 200 *μ*L of elution buffer (100 mM NaHCO_3_, 1% SDS), and then, 15 *μ*L of 5 M NaCl was added followed by incubation at 65°C for 4 hours. The DNA was purified by magnetic beads. The immunoprecipitated DNA and the input DNA were used as templates for quantitative fluorescence PCR using primers designed for the promoter regions of *SOX6*, *EIF2AK*, *BGLT3*, and *ZBTB7A4* genes. The primers used are listed in supplemental Table 3.

## 3. Results

### 3.1. Expression Changes of ANTXR1, *β*-Catenin, and *γ*-Globin during Erythroid Differentiation

**Figure 1:**
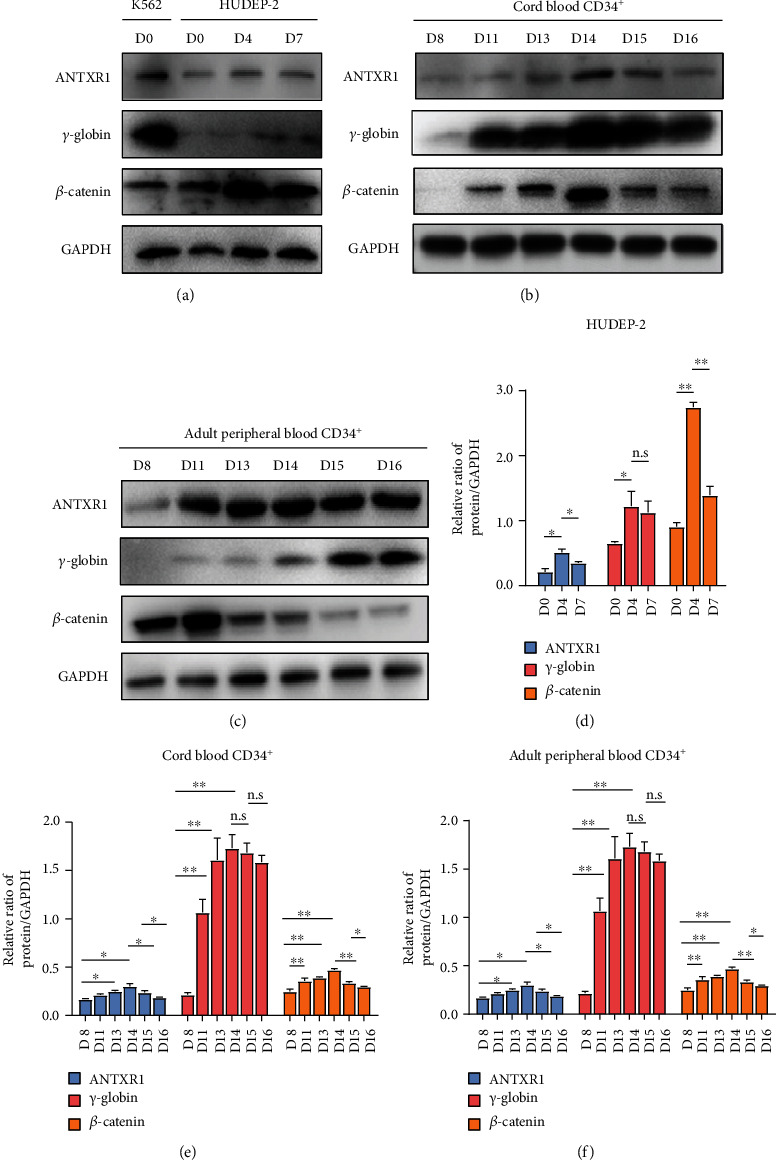
Expression of ANTXR1, *γ*-globin, and *β*-catenin during erythroid differentiation. (a) Western blot analysis was performed to detect the expression levels of ANTXR1, *γ*-globin, and *β*-catenin in K562 cells and HUDEP-2 cells at days 0, 4, and 7 of erythroid differentiation. (b) Western blot analysis for the expression of ANTXR1, *γ*-globin, and *β*-catenin in umbilical cord blood CD34^+^ cells during erythroid differentiation at days 8, 11, 13, 14, 15, and 16. (c) Western blot analysis for the expression of ANTXR1, *γ*-globin, and *β*-catenin in adult peripheral blood CD34^+^ cells during erythroid differentiation at days 8, 11, 13, 14, 15, and 16. (d) Protein quantification of the western blot in HUDEP-2 cells ANTXR1, *γ*-globin, and *β*-catenin at days 0, 4, and 7. (e) Quantification of western blot results of ANTXR1, *γ*-globin, and *β*-catenin in umbilical cord blood CD34^+^ cells at days 8, 11, 13, 14, 15, and 16. (f) Quantification of western blot results of ANTXR1, *γ*-globin, and *β*-catenin in adult peripheral blood CD34^+^ cells at days 8, 11, 13, 14, 15, and 16. These experiments *n* = 3. The error bar represents the SD. ^∗^*P* < 0.05 and ^∗∗^*P* < 0.01.

We examined the expression of ANTXR1, *β*-catenin, and *γ*-globin in K562 cells and the dynamic expression of these three proteins during erythroid differentiation of CB CD34^+^ cells, AB CD34^+^ cells, and HUDEP-2 cells. Western blot analysis showed that all three proteins were expressed in K562 cells (Figure 1(a)). The protein levels of ANTXR1, *β*-catenin, and *γ*-globin gradually increased during erythroid differentiation of CB and AB CD34^+^ cells, followed by a decrease after in the expression levels of ANTXR1 and *β*-catenin from days 8 to days 16, whereas the expression levels of *γ*-globin gradually increased and still maintain a high level of expression or slightly decreased. (Figures 1(b), 1(c), 1(e), and 1(f)). The kinetics of ANTXR1, *β*-catenin, and *γ*-globin expression during erythroid differentiation of HUDEP-2 cells (Figure 1(d)) were similar to those of CB CD34^+^ cells, with the highest expression by day 4 of differentiation. The above results indicated that the expression trends of ANTXR1 and *β*-catenin gradually decreased after initial rise and *γ*-globin with the highest expression levels in the middle and late stages; their differentiation decreased until the erythrocytes denucleated to form mature erythrocytes.

### 3.2. ANTXR1 on Time-Dependent *γ*-Globin Expression

**Figure 2:**
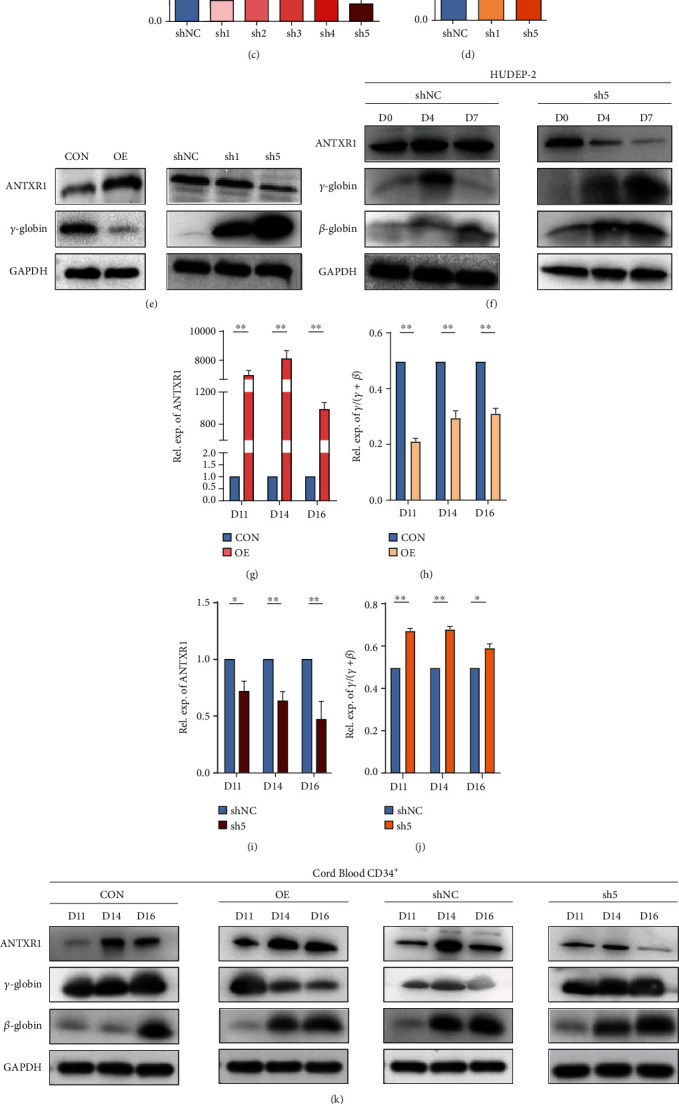
Effects of ANTXR1 overexpression or knockdown on *γ*-globin mRNA and protein levels. (a, b) *ANTXR1* was overexpressed using the PHAGE-fEF-1a-IRES-ZsGreen-2 vector in K562 cells, while cells transfected with the empty vector served as controls. ANTXR1 and *γ*-globin mRNA levels were measured by qRT-PCR. (c) In K562 cells, ANTXR1-sh1-5 was separately inserted into the pSIH1-H1-copGFPshRNA vector to test the knockdown efficiency of *ANTXR1*. Of the five shRNA vectors, ANTXR1-sh1 and ANTXR1-sh5 had the most significant knockdown efficiencies of 82% and 88%, respectively. (d) After the K562 cells were infected with ANTXR1-sh1, ANTXR1-sh5, and control shNC, respectively, the mRNA levels of ANTXR1 and *γ*-globin were determined using qRT-PCR. ANTXR1-sh5 had a greater effect on *γ*-globin expression than ANTXR1-sh1. (e) Western blot analysis was used to detect ANTXR1 and *γ*-globin levels after infection of K562 cells with *ANTXR1* overexpression vectors and interference vectors (ANTXR1-sh1 and ANTXR1-sh5). (f) At days 0, 4, and 7 of erythroid differentiation after infection of HUDEP-2 cells with ANTXR1-sh5 and shNC vectors, ANTXR1,*γ*-globin, and *β*-globin expression was determined by western blotting. (g, h) After infection of umbilical cord blood CD34^+^ cells with overexpression of the *ANTXR1* vector, the mRNA levels of *ANTXR1* and *γ*/*γ*+*β* globin ratio at days 11, 14, and 16 of erythroid differentiation were determined by qRT-PCR. (i, j) After infection of umbilical cord blood CD34^+^ cells with ANTXR1-5 and shNC vectors, the mRNA levels of *ANTXR1* and *γ*/*γ*+*β* globin ratio at days 11, 14, and 16 of erythroid differentiation were determined by qRT-PCR. (k) After infection of umbilical cord blood CD34^+^ cells with the *ANTXR1* overexpression vector and interference vector, the protein levels of ANTXR1,*γ*-globin, and *β*-globin at days 11, 14, and 16 of erythroid differentiation were detected by western blotting. (l) Umbilical cord blood CD34^+^ cells interfered with *ANTXR1* and were cultured under conditions that promoted erythroid maturation. The cells were collected at days 11, 14, and 16, respectively. Flow cytometry was used to detect HbF-immunostained cells (F cells). These experiments were repeated three times. The error bar represents the SD. ^∗^*P* < 0.05 and ^∗∗^*P* < 0.01. NC designates the negative control.

We transfected lentiviral vectors overexpressing and interfering with the *ANTXR1* gene in K562, CB CD34^+^, and HUDEP-2 cells, respectively. In K562 cells transfected with *ANTXR1* overexpression vectors, both the mRNA and protein levels of *γ*-globin genes were decreased significantly compared with the control group (Figures 2(a), 2(b), and 2(e)). After transfection with five kinds of vectors that interfered with *ANTXR1* (ANTXR-shRNA1-5) in K562 cells, the highest knockdown efficiency was observed for ANTXR1-sh1 and ANTXR1-sh5, which decreased the mRNA expression of *ANTXR1* by 82% and 88%, respectively, and significantly increased the mRNA and protein levels of *γ*-globin genes in the two groups of cells, especially ANTXR1-sh5 (Figures 2(c)–2(e)). Therefore, in subsequent experiments with CB CD34^+^ and HUDEP-2 cells, we only selected ANTXR1-sh5 to interfere with the *ANTXR1* gene.

During erythroid differentiation of CB CD34^+^ cells, we found that *ANTXR1* overexpression significantly reduced the *γ*/*γ*+*β* globin ratio, as measured by RT-qPCR (Figures 2(g) and 2(h)). Compared with the control group, *ANTXR1* overexpression was found to reduce the protein levels of *γ*-globin genes but not of *β*-globin genes at days 11, 14, and 16, as determined through western blotting (Figure 2(k)). By contrast, interference with *ANTXR1* significantly increased the *γ*/*γ*+*β* globin ratio, as measured through RT-qPCR (Figures 2(i) and 2(j)). Interference with *ANTXR1* increased the *γ*-globin protein levels without increasing the *β*–globin protein levels compared with the control group (Figure 2(k)). Similarly, in erythroid differentiation of HUDEP-2 cells, *γ*-globin expression was found to increase more significantly than the *β*-globin expression on the 7th day after ANTXR1 interference (Figure 2(f)). The flow cytometric results also showed an increase in the percentage of HbF-positive cells during erythroid differentiation of CB CD34^+^ cells that interfered with *ANTXR1* (Figure 2(l)). These results suggest that ANTXR1 has a negative regulatory effect on *γ*-globin gene expression in the middle and late stages of erythroid differentiation.

### 3.3. ANTXR1 Regulated the Activity of Wnt/*β*-Catenin

**Figure 3:**
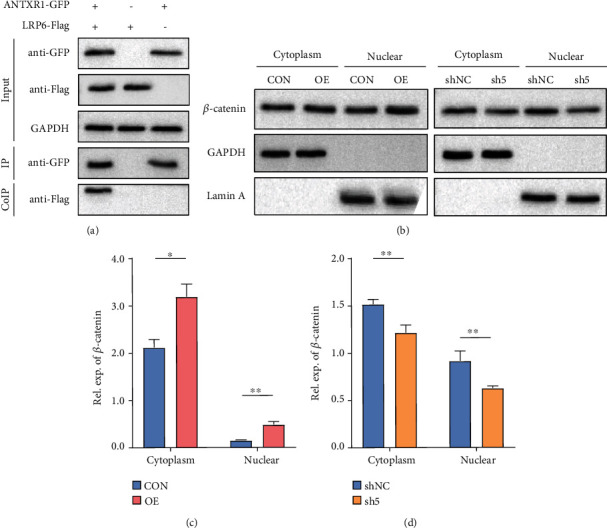
ANTXR1 activates the Wnt/*β*-catenin signaling pathway by promoting *β*-catenin into the nucleus. (a) ANTXR1 interacted with LRP6 shown by the Co-IP assay that the lysates from the K562 cells transfected with the ANTXR1–GFP and LRP6-Flag plasmids by using GFP- and Flag-targeting antibodies. (b) *β*-Catenin expression in the cytoplasm and nucleus was detected by western blot analysis after K562 cells were transfected with the ANTXR1 vector, control vector, ANTXR1-shRNA vector, or NC-shRNA vector. (c, d) Quantification of western blots. The error bar represents the SD.^∗^*P* < 0.05 and ^∗∗^*P* < 0.01. NC designates the negative control.

Previous studies have reported that ANTXR1 can facilitate the activation of the canonical Wnt signaling pathway in a variety of cancers [19]. To further verify whether ANTXR1 has a regulatory effect on the Wnt/*β*-catenin signaling pathway in hematological functions with the STRING database 9.0 (https://string-db.org), we found that the protein LRP6 was most likely to interact with ANTXR1. Then, we conducted Co-IP experiments to determine whether ANTXR1 interacts with LRP6 and activates the Wnt/*β*-catenin pathway. Following K562 cell transfection with the ANTXR1–GFP and LRP6-Flag fusion plasmids, both GFP and Flag were immunoprecipitated when the GFP antibody was used for IP, indicating that ANTXR1 and LRP6 interacted at the protein level (Figure 3(a)).

**Figure 4:**
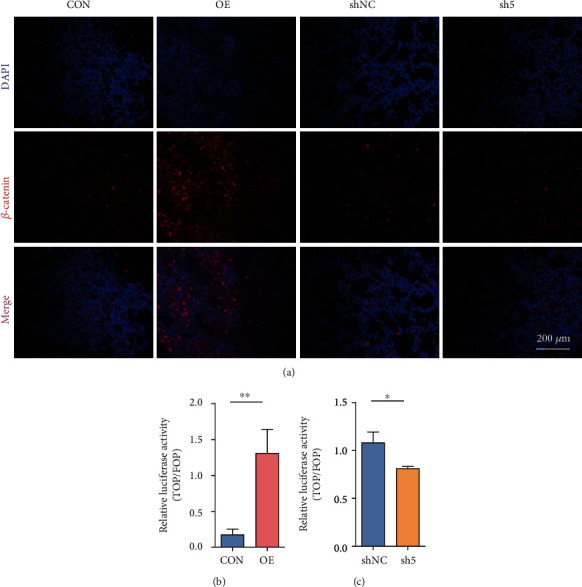
ANTXR1 activates the Wnt/*β*-catenin signaling pathway by promoting *β*-catenin into the nucleus. (a) The expression of *β*-catenin in K562 cells after overexpression or knockdown of ANTXR1 was detected by immunofluorescence with a confocal laser scanning microscope. The color “red” represents *β*-catenin, and “blue” represents nuclei stained by DAPI. The merged images indicate the localization of *β*-catenin in both the nuclei and cytoplasm. (b, c) The TOP-/FOP-flash luciferase assay shows the activity of the Wnt/*β*-catenin signaling pathway in 293T cells overexpressing or knocking down the *ANTXR1* gene. Experiments *n* = 3. The error bar represents the SD.^∗^*P* < 0.05 and ^∗∗^*P* < 0.01. NC designates the negative control.

Next, we examined the changes in *β*-catenin expression in the nucleus and cytoplasm of K562 cells after overexpression and knockdown of *ANTXR1* using an immunofluorescence assay and a nuclear and cytoplasmic protein separation assay. The immunofluorescence results showed that nuclear *β*-catenin expression was increased after *ANTXR1* overexpression and decreased after *ANTXR1* knockdown compared with the control (Figure 4(a)). The results of nuclear and cytoplasmic protein separation experiments showed that *β*-catenin protein levels increased 3.2-fold in the nucleus but only 1.74-fold in the cytoplasm after *ANTXR1* overexpression, while *β*-catenin protein levels were reduced by 32% in the nucleus but only by 23.8% in the cytoplasm after *ANTXR1* knockdown (Figures 3(b)–3(d)). Then, we found that overexpression of *ANTXR1* in K562 cells increased TOP/FOP luciferase activity by 6.38-fold, whereas knockdown of *ANTXR1* decreased TOP/FOP luciferase activity by 1.38-fold compared with the control (Figures 4(b) and 4(c)). These results indicate that ANTXR1 can promote *β*-catenin nuclear entry and activate the Wnt/*β*-catenin signaling pathway.

### 3.4. ANTXR1 Regulates *γ*-Globin Expression by Affecting the Wnt/*β*-Catenin Pathway

**Figure 5:**
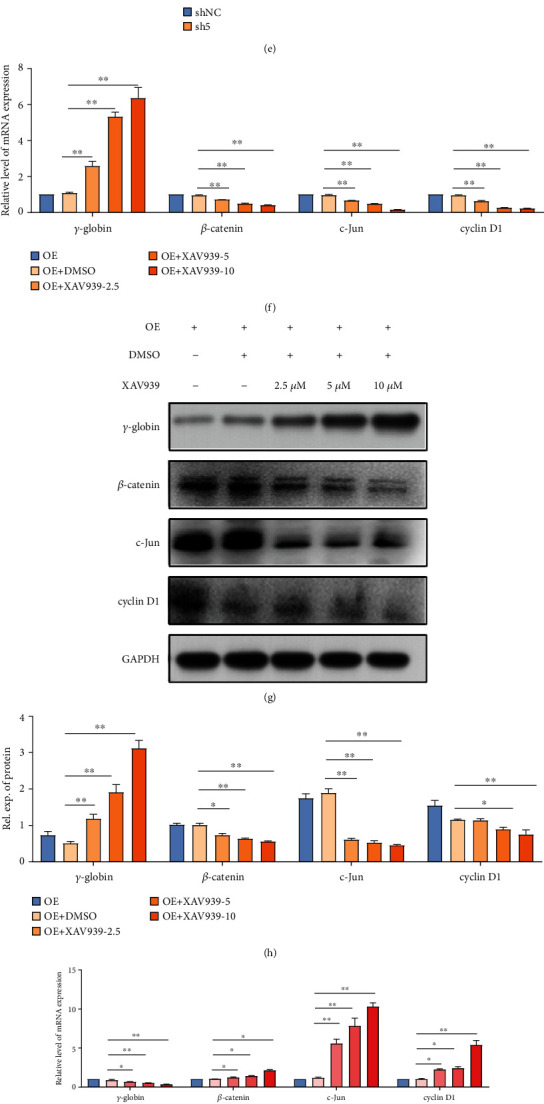
Effect of ANTXR1 on the expression of key molecules of the Wnt/*β*-catenin pathway. (a) The mRNA expression levels of LRP6, *β*-catenin, c-Jun, and cyclin D1 were detected by qRT-PCR after overexpression of *ANTXR1* in K562 cells. (b) The mRNA expression levels of LRP6, *β*-catenin, c-Jun, and cyclin D1 were detected by qRT-PCR, after K562 cells were transfected with ANTXR1-sh5. (c) The protein levels of LRP6, *β*-catenin, c-Jun, and cyclin D1 were measured by western blotting after K562 cells were overexpressed or knocked down by *ANTXR1*. (d, e) Quantification of the western blots is shown in (c). (f, g) The mRNA and protein expression levels of *γ*-globin, LRP6, *β*-catenin, c-Jun, and cyclin D1 were detected by qRT-PCR and western blotting, respectively, with K562 cells overexpressing *ANTXR1* treated with 2.5, 5, and 10 *μ*mol/L of XAV939 for 24 h. (h) Quantification of the western blots shown in (g). (i, j) The mRNA and protein expression levels of *γ*-globin, LRP6, *β*-catenin, c-Jun, and cyclin D1 were detected by qRT-PCR and western blotting, respectively, with K562 cells interfering with ANTXR1-sh5 which were treated with 5, 20, and 40 mmol/L of LiCl for 24 h. (k) Quantification of the western blots shown in (j). These experiments *n* = 3. The error bar represents the SD. ^∗^*P* < 0.05, ^∗∗^*P* < 0.01. NC: negative control.

The above results prompted us to investigate whether ANTXR1 affects key molecules in the Wnt/*β*-catenin signaling pathway, thereby affecting *γ*-globin expression. The results of this study showed that the mRNA and protein levels of LRP6, *β*-catenin, and downstream proteins c-Jun and cyclin D1, which are key molecules of the Wnt/*β*-catenin signaling pathway, were significantly higher than those in the control group (Figures 5(a), 5(c), and 5(d)) after overexpression of *ANTXR1* in K562 cells, while the mRNA and protein levels of these key molecules were significantly lower after knockdown of *ANTXR1* (Figures 5(b), 5(c), and 5(e)).

To further verify whether the Wnt/*β*-catenin signaling pathway is involved in the regulation of *γ*-globin by ANTXR1, different concentrations of the Wnt/*β*-catenin signaling pathway inhibitor XAV939 as well as the activator LiCl were added to the medium to block and activate the Wnt/*β*-catenin signaling pathway, respectively. The results showed that the addition of different concentrations of XAV939 significantly reversed the expression of the repressed mRNA and protein levels of *γ*-globin genes in K562 cells stably transfected with *ANTXR1*. Meanwhile, the mRNA and protein expression levels of *β*-catenin, c-Jun, and cyclin D1 after the addition of XAV939 were also significantly lower than those of the control group in a dose-dependent manner (Figures 5(f)–5(h)). In contrast, the addition of different concentrations of the activator LiCl to K562 cells stably knocked down for *ANTXR1* significantly inhibited the otherwise increased *γ*-globin expression. Meanwhile, the mRNA and protein expression levels of *β*-catenin, c-Jun, and cyclin D1 were significantly higher after addition of LiCl than those in the control group (Figures 5(i)–5(k)). The above results showed that the effects of overexpression and knockdown of *ANTXR1* on the Wnt/*β*-catenin signaling pathway and *γ*-globin expression could be reversed by the inhibitor XAV939 and the activator LiCl, respectively, indicating that ANTXR1 may regulate *γ*-globin expression through the Wnt/*β*-catenin signaling pathway.

### 3.5. Overexpression of ANTXR1 Promoted the Combination of c-Jun and SOX6 Gene

**Figure 6:**
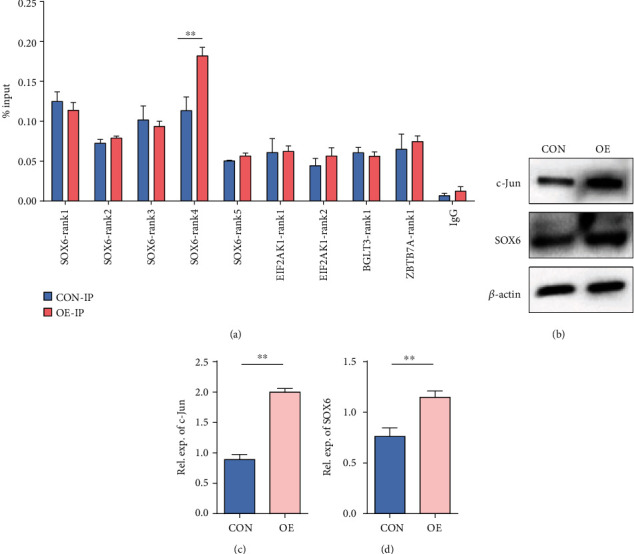
Overexpression of *ANTXR1* promoted the combination of c-Jun and *SOX6*. (a) ChIP-qPCR assays were performed using K562 cells expressing *ANTXR1*, while an empty vector served as a control. The immunoprecipitated DNA of *SOX6*, *EIF2AK*, *BGLT3*, and *ZBTB7A 4* binding site to c-Jun, as well as input DNA, were used as templates for quantitative fluorescence PCR. The enrichments were quantified by ChIP-qPCR and normalized by comparison to input DNA (% input). (b) After overexpression of *c-Jun* in K562 cells, the protein levels of c-Jun and SOX6 were measured by western blotting. (c, d) Quantification of the western blot. The experiments *n* = 3. The error bar represents the SD. ^∗∗^*P* < 0.01.

Since ANTXR1 enhanced Wnt/*β*-catenin signaling pathway activity, the mRNA and protein expression of its downstream target gene *c-Jun* was significantly increased and c-Jun is a transcription factor that belongs to the activator protein-1 family of proteins. Thus, we speculate that ANTXR1 may indirectly regulate *γ*-globin expression by activating c-Jun, thereby affecting the expression of genes bound to it. To verify this hypothesis, we first analyzed the data from the ChIP-seq public database CR Cistrome (http://cistrome.org/db/#/) and UCSC Genome Browser database, searching for genes that inhibit *γ*-globin expression with c-Jun binding sites in the transcriptional regulatory regions. The results showed that four genes (*SOX6*, *EIF2AK*, *BGLT3*, and *ZBTB7A*) met the search criteria, of which *SOX6* has five sites (SOX6-rank1-5) (Supplemental Figure 1), *EIF2AK2* has two sites (EIF2AK-rank1-2), and both *BGLT3* and *ZBTB7A* have one site (Supplemental Table 4). Next, the results of the ChIP-qPCR experiments showed that among the nine sites, only SOX6-rank4 showed a significant increase in binding ability to c-Jun in K562 cells stably overexpressing the *ANTXR1* gene compared with the control group (Figure 6(a)). We further overexpressed *c-Jun* in K562 cells and found a 1.5-fold increase in SOX6 protein levels compared with those in the control (Figures 6(b)–6(d)). Therefore, it can be inferred that ANTXR1 promotes Wnt/*β*-catenin signaling pathway activity, which initiates the expression of c-Jun and then promotes SOX6 expression, which could be one of the important repressors of *γ*-globin expression.

## 4. Discussion

Reactivation of silenced fetal hemoglobin as a therapeutic strategy has been shown promising. Although ANTXR1 is a candidate as an additional HbF modulator, the specific molecular mechanism remains elusive at present. In this study, we investigated, for the first time, the membrane protein ANTXR1 that regulates *γ*-globin expression through the Wnt signaling pathway.

During erythroid differentiation of three cell lines (CB CD34^+^, AB CD34^+^, and HUDEP-2 cells), we found that the expression levels of these three proteins gradually increased during cell differentiation and were highest in the middle and late stages of differentiation, followed by a decrease in the expression of ANTXR1 and *β*-catenin, whereas the expression of *γ*-globin did not change, still maintaining a high level of expression (CB CD34^+^, AB CD34^+^, and HUDEP-2). These results are similar to those of previous studies that iPSCs were used in immunofluorescence costaining [13], suggesting that ANTXR1, *β*-catenin, and *γ*-globin are associated during erythroid differentiation. Furthermore, we found that the expression of *γ*-globin was decreased and increased after overexpression and knockdown of *ANTXR1* in K562, CB CD34^+^, and HUDEP-2 cells, respectively, which further indicated that ANTXR1 negatively regulates the expression of *γ*-globin.

Because ANTXR1 is a membrane protein, we hypothesize that it may regulate the expression of *γ*-globin genes through certain mediators or signaling pathways. Cell signaling pathways also play an important role in the regulation of the expression of *γ*-globin genes [20–22]. Some inducers of *γ*-globin genes regulate their expression through different cell signaling pathways; for example, the p38 MAPK signaling pathway plays an important role which is related to the regulation of *γ*-globin in K562 cells [21]. Our data showed that *β*-catenin, an important factor in the Wnt/*β*-catenin pathway, is consistent with the expression changes of ANTXR1 in erythroid differentiation, suggesting that the Wnt/*β*-catenin pathway may play a role in the regulation of *γ*-globin expression by ANTXR1.

Wnt/*β*-catenin is a canonical Wnt signaling pathway that is highly conserved during evolution and regulates and controls numerous processes of life activities [23]. Previous studies have shown that activation of the Wnt/*β*-catenin pathway maintains hematopoietic cells in an immature state even though it promotes the proliferation of hematopoietic stem cells, while inhibition of this pathway predisposes hematopoietic stem cells to differentiation [24–26]. Wnt/*β*-catenin signaling regulates various cellular components in the hematopoietic stem cell microenvironment to maintain the stability of the hematopoietic microenvironment [27]. During erythroid differentiation of hematopoietic stem cells, *γ*-globin also shows dynamic changes, so it is speculated that the Wnt/*β*-catenin signaling pathway is not only involved in the proliferation and differentiation process of hematopoietic stem cells but is also involved in the regulation of *γ*-globin expression.

It is well-known that ANTXR1 affects the activity of the Wnt/*β*-catenin signaling pathway through its action on LRP6, thus exerting the corresponding biological function [16, 17]. We, therefore, speculate that ANTXR1 may be involved in regulating *γ*-globin expression by activating the Wnt/*β*-catenin signaling pathway through LRP6. To confirm this hypothesis, the subsequently performed Co-IP analysis indicated that there was an interaction betweenANTXR1 and LRP6, likely through LRP6 interaction with ANTXR1 in its extracellular domain. However, it is not clear whether this effect is direct or indirect [15].

Next, we overexpressed or knocked down *ANTXR1* in K562 cells, with the results showing that the expression of LPR6 and *β*-catenin was upregulated and downregulated, respectively, while promoting *β*-catenin nuclear entry or exit, respectively. This phenomenon was consistent with changes in the enhanced and decreased activity of the Wnt/*β*-catenin signaling pathway. However, it remains unclear why ANTXR1 can promote the expression of LPR6 and *β*-catenin.

To further confirm the role of Wnt/*β*-catenin in the regulation of *γ*-globin by ANTXR1, we designed rescue experiments. The results showed that the addition of XAV939, an inhibitor of the Wnt/*β*-catenin signaling pathway for K562 cells stably transfected with the *ANTXR1* gene, significantly reversed the mRNA and protein expression of *γ*-globin that had been inhibited, while the expression of *β*-catenin and c-Jun and cyclin D1, downstream proteins of the Wnt/*β*-catenin pathway, was also significantly downregulated. In contrast, when LiCl, an activator of the Wnt/*β*-catenin signaling, was added after interfering with *ANTXR1* in K562 cells, otherwise, increased expression of *γ*-globin could be significantly inhibited, and the expression of the *β*-catenin, c-Jun, and cyclin D1 proteins could be significantly upregulated. These results indicate that the Wnt/*β*-catenin signaling pathway plays an important role in the regulation of *γ*-globin expression by ANTXR1.

Upon activation of the Wnt/*β*-catenin signaling pathway, the key player, *β*-catenin translocates from the cytoplasm to the nucleus and forms a transcriptional activation complex with the transcription factor T cytokine/lymphoid enhancer factor (TCF/LEF), thereby activating downstream target genes [28]. Our results showed that the expression of c-Jun, a downstream transcription factor of the Wnt/*β*-catenin pathway, was consistent with the expression changes of ANTXR1 after overexpression or knockdown of the *ANTXR1* gene in K562 cells. ChIP-qPCR results showed that the binding ability of the rank4 site in the transcriptional regulatory region of the *SOX6* gene to c-Jun was significantly increased after overexpression of *ANTXR1* in K562 cells. We also found that SOX6 protein expression was increased significantly after overexpression of the *c-Jun* gene in K562 cells, indicating that the transcription factor c-Jun initiated the transcription of *SOX6*. SOX6 has been demonstrated to act synergistically with BCL11A and GATA1 to mediate the silencing of the *γ*-globin genes [4, 29]. Therefore, the regulation of *γ*-globin expression by ANTXR1 is achieved ultimately through SOX6. In addition, we also found that ANTXR1 correlated with the expression levels of *γ*-globin gene expression regulators such as BCL11A and KLF1 (Supplemental Figure 2), although the specific mechanism was unclear and needs to be further investigated.

**Figure 7:**
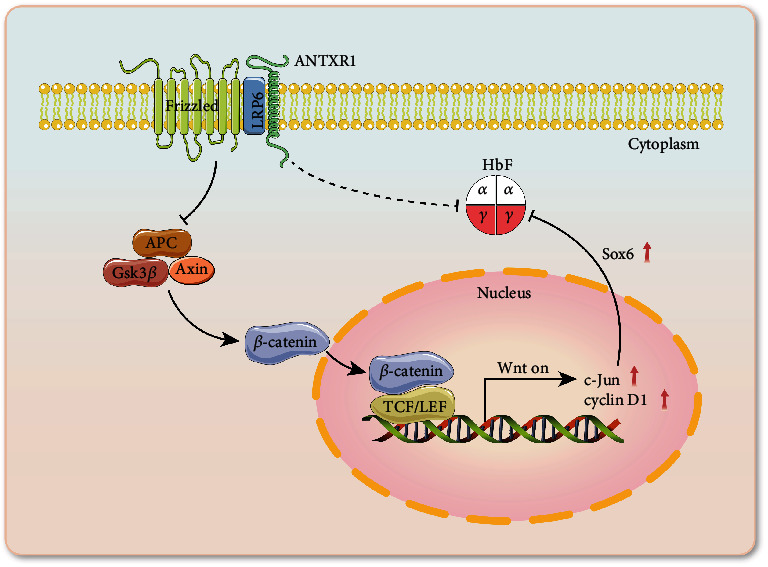
Molecular mechanisms by which ANTXR1 regulates *γ*-globin expression via the Wnt/*β*-catenin signaling pathway. The interaction of ANTXR1 with LRP6 may inhibit the activity of the *β*-catenin destructive complex consisting of Axin, APC, and Gsk3 via Frizzled. *β*-Catenin is then free to translocate to the nucleus where it binds to the TCF/LEF transcription factors, thereby promoting the transcription of target gene *c-Jun*, which, in turn, activates the expression of SOX6, a repressor of *γ*-globin genes, thereby inhibiting the expression of *γ*-globin genes.

In summary, the molecular mechanism by which ANTXR1 regulates *γ*-globin expression is mainly through interacting with the membrane protein LRP6 and activating the Wnt/*β*-catenin signaling pathway, which initiates the expression of the downstream transcription factor c-Jun, which, in turn, promotes SOX6 expression, thereby silencing the expression of *γ*-globin (Figure 7). However, Wnt/*β*-catenin involved signaling is a complex. The regulation of *γ*-globin expression by ANTXR1 may also involve multiple levels, including effects on the expression of key regulators of *γ*-globin genes such as BCL11A and KLF1. The results of this study enrich our knowledge of the regulatory network controlling *γ*-globin expression and may provide a new intervention target for the treatment of *β*-hemoglobinopathies. Finally, it is worthwhile to mention the strong evidence that patients with hemoglobinopathy have an increased risk of COVID-19-related hospital admissions; large cohort studies in the UK and US have reported a 2- to 7-fold increased risk of COVID-19-related hospital admission for patients with SCD relative to the general population [30].

## Data Availability

The data used to support the findings of this study are included within the article.

## Abbreviations

HbF: Fetal hemoglobin
SCD: Sickle cell disease
ANTXR1: Anthrax toxin receptor 1
TEM8: Tumor endothelial marker 8
ECs: Endothelial cells
SNP: Single nucleotide polymorphism
LRP: LDL-receptor-related protein
FBS: Fetal bovine serum
FACS: Fluorescence-activated cell sorting
CB: Cord blood
AB: Adult peripheral blood
NC-shRNA: Negative control shRNA
CTNNB1: *β*-Catenin gene
CCND1: Cyclin D1gene
Co-IP: The coimmunoprecipitation.

## Supplementary Materials

Supplementary Materials Supplementary Figure S1: binding sites of Jun to *SOX6*. The sites were obtained from the UCSC Genome Browser database. Among the six different *SOX6* transcripts, CHIP data showed the binding site map of Jun to *SOX6*. The green boxes represent the binding sites of Jun to *SOX6* in K562 cells, named rank1, rank2, rank3, rank4, and rank5, respectively. Shown within the red box are six different transcripts of the *SOX6* gene. Figure S2: effects of *ANTXR1* overexpression or knockdown on *BCL11A* and *KLF1* mRNA levels in K562 cells. (A, B) *ANTXR1* was overexpressed in K562 cells using a pHAGE-fEF-1a-IRES-ZsGreen-2 vector. Cells transfected with an empty vector served as controls. The effects of *ANTXR1* overexpression on *BCL11A* and *KLF1* expression measured by RT-PCR. Overexpression of *ANTXR1* in K562 cells significantly increased *BCL11A* and *KLF1* expression compared to that observed in the control group; (C, D) ANTXR1-sh5 or NC-shRNA vector-transfected K562 cells. The effects of *ANTXR1* knockdown on *BCL11A* and *KLF1* expression measured by RT-qPCR. Knockdown of *ANTXR1* in K562 cells significantly decreased the expression of *BCL11A* and *KLF1* compared with that observed in the control group. Three independent assays were performed with the error bar representing the SD. ^∗^*P* < 0.05 and ^∗∗^*P* < 0.01. Table S1: sequences of the five designed shRNAs targeting the ANTXR1 gene and NC-shRNA sequences. Table S2: list of primers used for RT-qPCR assays. Table S3: sequences of primer pairs for ChIP-qPCR analysis. Table S4: the positions of the binding sites of SOX6, EIF2AK, BGLT3, and ZBTB7A with Jun.
