# Supplementary material for: Transmembrane Protein ANTXR1 Regulates γ-Globin Expression by Targeting the Wnt/β-Catenin Signaling Pathway
Source: J Immunol Res. 2022 Jul 30;2022:8440422. doi: 10.1155/2022/8440422 (PMC9356848; doi:10.1155/2022/8440422)
Supplement: Supplementary Materials — Supplementary Figure S1: binding sites of Jun to SOX6. The sites were obtained from the UCSC Genome Browser database. Among the six different SOX6 transcripts, CHIP data showed the binding site map of Jun to SOX6. The green boxes represent the binding sites of Jun to SOX6 in K562 cells, named rank1, rank2, rank3, rank4, and rank5, respectively. Shown within the red box are six different transcripts of the SOX6 gene. Figure S2: effects of ANTXR1 overexpression or knockdown on BCL11A and KLF1 mRNA levels in K562 cells. (A, B) ANTXR1 was overexpressed in K562 cells using a pHAGE-fEF-1a-IRES-ZsGreen-2 vector. Cells transfected with an empty vector served as controls. The effects of ANTXR1 overexpression on BCL11A and KLF1 expression measured by RT-PCR. Overexpression of ANTXR1 in K562 cells significantly increased BCL11A and KLF1 expression compared to that observed in the control group; (C, D) ANTXR1-sh5 or NC-shRNA vector-transfected K562 cells. The effects of ANTXR1 knockdown on BCL11A and KLF1 expression measured by RT-qPCR. Knockdown of ANTXR1 in K562 cells significantly decreased the expression of BCL11A and KLF1 compared with that observed in the control group. Three independent assays were performed with the error bar representing the SD. ∗P < 0.05 and ∗∗P < 0.01. Table S1: sequences of the five designed shRNAs targeting the ANTXR1 gene and NC-shRNA sequences. Table S2: list of primers used for RT-qPCR assays. Table S3: sequences of primer pairs for ChIP-qPCR analysis. Table S4: the positions of the binding sites of SOX6, EIF2AK, BGLT3, and ZBTB7A with Jun. [file 8440422.f1.docx]

**Supplemental Materials**

**Supplemental Methods**

**Induced CD34+ cell culture**

Isolated CD34^+^ cells (STEMCELL, #17896，#17856) were first plated in StemSpan SFEM II (STEMCELL Technologies) supplemented with 50 ng/mL SCF, 50 ng/mL Flt3 ligand, 50 ng/mL TPO and 2% penicillin-streptomycin for 7 days according to the manufacturer's instructions. On day 7, the cells were cultured to promote erythroid differentiation using conditions modified from those reported previously^1^. The cells were inoculated for 7 days at a density of 5 x 10^5^/mL in StemSpan SFEM II medium containing 10 ng/mL SCF，10 ng/mL IL-3，3 IU/mL EPO，and 2% penicillin-streptomycin, followed by the addition of 10 ng /mL SCF, 3 IU/mL EPO and 2% penicillin-streptomycin and culture for a further 3 days. 1 IU/mL EPO and 2% penicillin-streptomycin were then added continually to the SFEM Ⅱ medium for a further 6 days, resulting in a total incubation time of 16 days.

**Induced HUDEP-2 cell culture**

During the proliferation phase of HUDEP-2 cells, the medium was changed every 3-4 days and the cell density kept below 800,000 cells/mL to maintain the optimal growth rate. HUDEP-2 cell expansion was performed in StemSpan SFEM II (SFEMCELL Technologies) containing 2% penicillin-streptomycin solution (10,000 U/mL stock), 50 ng/mL recombinant human stem cell factor (SCF), 3 IU/mL Epoetin alfa (Epogen, Amgen), 0.4 μg/mL dexamethasone, and 1μg/mL doxycycline. Culture of the HUDEP Cells for erythroid differentiation involved：1. Transfer of the cells to the erythroid differentiation medium (EDM) containing Iscove’s modified Dulbecco’s medium (IMDM), 2% penicillin-streptomycin solution (10,000 U/mL stock concentration), 500 μg/mL human holo-transferrin, 10 μg/mL recombinant human insulin solution, 3 IU/mL heparin, 3% inactivated human plasma, and 3 IU/mL Epoetin alfa (Epogen, Amgen). 2. Culture for 4 days in EDM containing 100 ng/mL SCF and 1 μg/mL doxycycline. 3. Culture for 3 days in EDM containing 1 μg/mL doxycycline^2^.

**Supplemental Data References**

1. Vathipadiekal V., Farrell J.J., Wang, S. et al, A candidate transacting modulator of fetal hemoglobin gene expression in the Arab-Indian haplotype of sickle cell anemia, Am J Hematol 91 (2016) 1118-1122.

2. Vinjamur D.S., Bauer D.E.. Growing and genetically manipulating human umbilical cord blood-derived erythroid progenitor (HUDEP) cell lines. Methods Mol Biol 1698 (2018) 275-284.

**Supplemental Table and Figures**

**Table S1.** Sequences of the five designed shRNA targeting the ANTXR1 gene and NC-shRNA sequences

| ANTXR1-sh1 | **Forward primer**:  GATCCGATTGCGGACAGTAAGGATCTTCCTGTCAGA ATCCTTACTGTCCGCAATCTTTTTG  **Reverse primer**:  AATTCAAAAAGATTGCGGACAGTAAGGATTCTGACAGGAAGATCCTTACTGTCCGCAATCG |
| --- | --- |
| ANTXR1-sh2 | **Forward primer**:  GATCCGCTGAACCATCCACCATATGTCTTCCTGTCAGAACATATGGTGGATGGTTCAGCTTTTTG  **Reverse primer**:  AATTCAAAAAGCTGAACCATCCACCATATGTTCTGACAGGAAGACATATGGTGGATGGTTCAGCG |
| ANTXR1-sh3 | **Forward primer**:  GATCCTAATAGGTCTCGAGACTTCCTGTCAGATCTCGAGACCTATTATTTTTG  **Reverse primer**:  AATTCAAAAATAATAGGTCTCGAGATCTGACAGGAAGTCTCGAGACCTATTAG |
| ANTXR1-sh4 | **Forward primer**:  GATCCCCGAGGAACAACCTTAATGAACTTCCTGTCAGATTCATTAAGGTTGTTCCTCGGTTTTTG  **Reverse primer**:  AATTCAAAAACCGAGGAACAACCTTAATGAATCTGACAGGAAGTTCATTAAGGTTGTTCCTCGGG |
| ANTXR1-sh5 | **Forward primer**:  GATCCCCCACAGTTGAGAATGTCCTTCTTCCTGTCAGAAAGGACATTCTCAACTGTGGGTTTTTG  **Reverse primer**:  AATTCAAAAACCCACAGTTGAGAATGTCCTTTCTGACAGGAAGAAGGACATTCTCAACTGTGGGG |
| NC-shRNA | **Forward primer**:  GATCCTTCTCCGAACGTGTCACGTCTTCCTGTCAGA ACGTGACACGTTCGGAGAATTTTTG  **Reverse primer**:  AATTCAAAAATTCTCCGAACGTGTCACGTTCTGACAGGAAGACGTGACACGTTCGGAGAAG |

**Table S2.** List of primers used for RT-qPCR assays

| GAPDH | **Forward primer** | GCACCGTCAAGGCTGAGAAC |
| --- | --- | --- |
|  | **Reverse primer** | TGGTGAAGACGCCAGTGGA |
|  | | |
| ANTXR1 | **Forward primer** | CGGATTGCGGACAGTAAGGAT |
|  | **Reverse primer** | TCCTCTCACGACAACTTGAAATG |
|  | | |
| γ-globin | **Forward primer** | TTCACAGAGGAGGACAAGGCTAC |
|  | **Reverse primer** | GCAGAGGCAGAGGACAGGTT |
|  | | |
| LRP6 | **Forward primer** | ATTTAGATGGATCTTTACGA |
|  | **Reverse primer** | ATAATGAAGCGACTTGAAC |
|  | | |
| β-catenin | **Forward primer** | TATTACGACAGACTGCCTTCA |
|  | **Reverse primer** | CAGATAGCACCTTCAGCAC |
|  | | |
| c-Jun | **Forward primer** | ATGGAAACGACCTTCTATGACGA |
|  | **Reverse primer** | CGAGGTGAGGAGGTCCGAGT |
|  | | |
| cyclin D1 | **Forward primer** | GCTGCGAAGTGGAAACCATC |
|  | **Reverse primer** | CCTCCTTCTGCACACATTTGAA |
|  |  |  |
| β-globin | **Forward primer** | AGGAGAAGTCTGCCGTTACTG |
|  | **Reverse primer** | CCGAGCACTTTCTTGCCATGA |

**Table S3.** Sequences of primer pairs for ChIP-qPCR analysis

| SOX6-rank1 | **Forward primer** | TGTTGGTCCAAACTGAAAGTTG |
| --- | --- | --- |
|  | **Reverse primer** | TCTGTGGACAGTGGGTTCTG |
|  | | |
| SOX6-rank2 | **Forward primer** | TCACTCAGATCGGCTTCTCTC |
|  | **Reverse primer** | CACGTGACATATGCTGTGCTT |
|  | | |
| SOX6-rank3 | **Forward primer** | ACATGTCGAGCAGAACACCA |
|  | **Reverse primer** | TGGGTTTTATCCTCTGGGAAA |
|  | | |
| SOX6-rank4 | **Forward primer** | GTCCCACAATTACCCCATTG |
|  | **Reverse primer** | CAGGCTCCCTAGCAGTTGAG |
|  | | |
| SOX6-rank5 | **Forward primer** | GGGAGCTGCTTTCTTCCTTC |
|  | **Reverse primer** | ACCGAGCTAATCTGCCCTCA |
|  | | |
| EIF2AK1-rank1 | **Forward primer** | CGCCTCATCATTTCATTCCT |
|  | **Reverse primer** | CTTTGCAAGGGTGTGGTTTT |
|  | | |
| EIF2AK1-rank2 | **Forward primer** | TGCCCAGTCGGAAAGGAG |
|  | **Reverse primer** | TCCTTGCCCACAGCCATT |
|  | | |
| BGLT3-rank1 | **Forward primer** | TGATGGTGTTACGGACCTAATG |
|  | **Reverse primer** | TGATGGGGATCTGCTCTTATG |
|  | | |
| ZBTB7A-rank1 | **Forward primer** | CCTGGAGGAATGTGAGCTGT |
|  | **Reverse primer** | ATCTGTCAAATGGGCAGAGG |

**Table S4.** The positions of the binding sites of SOX6, EIF2AK, BGLT3 and ZBTB7A with Jun

| **Gene** | **Transcript position** | **Strand** | **Binding site name** | **Position of binding site** | **Distance from binding site to the first base of exon1 (bp)** |
| --- | --- | --- | --- | --- | --- |
| SOX6 (transcript-1) | hg38 chr11:15966449-16341269 | - | rank1 | chr11:15969370-15969665 | 371752 |
|  |  |  | rank2 | chr11:16094068-16094363 | 247054 |
|  |  |  | rank3 | chr11:16359409-16359704 | -18288 |
|  |  |  | rank4 | chr11:16408749-16409044 | -67627 |
|  |  |  | rank5 | chr11:16604753-16605048 | -263631 |
| SOX6 (transcript-2) | hg38 chr11:15966449-16402867 | - | rank1 | chr11:15969370-15969665 | 433350 |
|  |  |  | rank2 | chr11:16094068-16094363 | 308652 |
|  |  |  | rank3 | chr11:16359409-16359704 | 43310 |
|  |  |  | rank4 | chr11:16408749-16409044 | -6029 |
|  |  |  | rank5 | chr11:16604753-16605048 | -202033 |
| SOX6 (transcript-3) | hg38 chr11:15966449-16476388 | - | rank1 | chr11:15969370-15969665 | 506871 |
|  |  |  | rank2 | chr11:16094068-16094363 | 382173 |
|  |  |  | rank3 | chr11:16359409-16359704 | 116831 |
|  |  |  | rank4 | chr11:16408749-16409044 | 67492 |
|  |  |  | rank5 | chr11:16604753-16605048 | -128512 |
| SOX6 (transcript-4) | hg38 chr11:15972699-16408884 | - | rank1 | chr11:15969370-15969665 | 439367 |
|  |  |  | rank2 | chr11:16094068-16094363 | 314669 |
|  |  |  | rank3 | chr11:16359409-16359704 | 49327 |
|  |  |  | rank4 | chr11:16408749-16409044 | -12 |
|  |  |  | rank5 | chr11:16604753-16605048 | -196016 |
| SOX6 (transcript-5) | hg38 chr11:15972708-16402846 | - | rank1 | chr11:15969370-15969665 | 433329 |
|  |  |  | rank2 | chr11:16094068-16094363 | 308631 |
|  |  |  | rank3 | chr11:16359409-16359704 | 43289 |
|  |  |  | rank4 | chr11:16408749-16409044 | -6050 |
|  |  |  | rank5 | chr11:16604753-16605048 | -202054 |
| SOX6 (transcript-6) | hg38 chr11:15972708-16402863 | - | rank1 | chr11:15969370-15969665 | 433346 |
|  |  |  | rank2 | chr11:16094068-16094363 | 308648 |
|  |  |  | rank3 | chr11:16359409-16359704 | 43306 |
|  |  |  | rank4 | chr11:16408749-16409044 | -6033 |
|  |  |  | rank5 | chr11:16604753-16605048 | -202037 |
| EIF2AK1 | chr7:6022247-6059175 | - | rank1 | chr7:6023981-6024276 | 35047 |
|  |  |  | rank2 | chr7:6056658-6056953 | 2370 |
| BGLT3 | chr11:5244554-5245546 | - | rank1 | chr11:5247542-5247837 | -2143 |
| ZBTB7A | chr19:4043303-4066899 | - | rank1 | chr19:4058326-4058621 | 8430 |
| ZBTB7A | chr19:4047742-4065732 | - | rank1 | chr19:4058326-4058621 | 7263 |


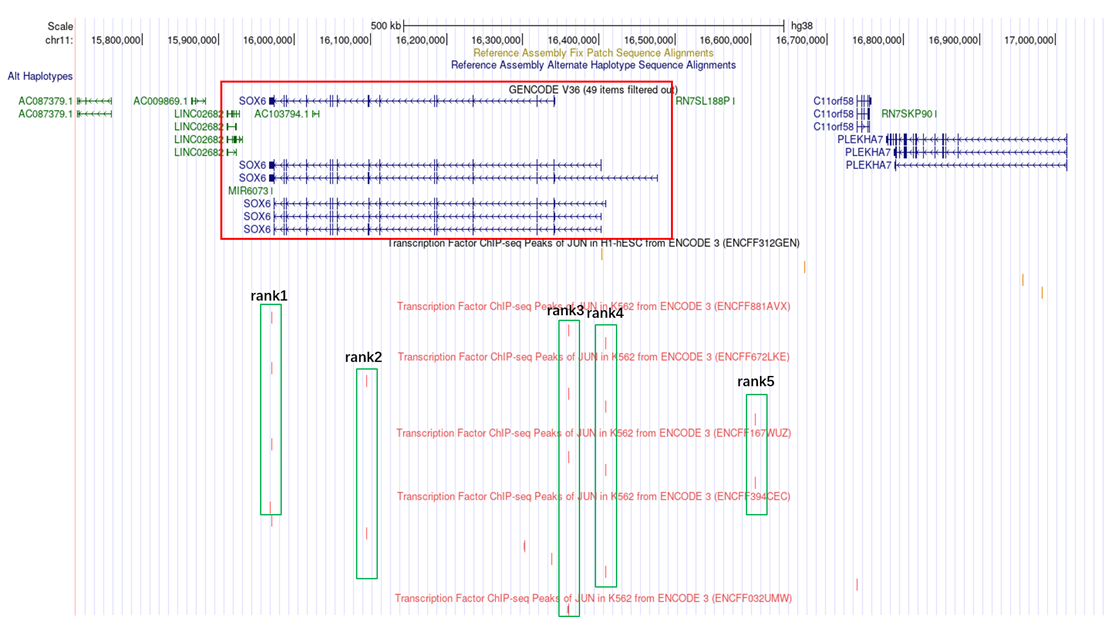


**Supplemental Figure 1: Binding sites of Jun to *SOX6*.** The sites were obtained from The UCSC Genome Browser database. Among the six different *SOX6* transcripts, CHIP data showed the binding site map of Jun to *SOX6*. The green boxes represent the binding sites of Jun to *SOX6* in K562 cells, named rank1, rank2, rank3, rank4, and rank5, respectively. Shown within the red box are six different transcripts of the *SOX6* gene.


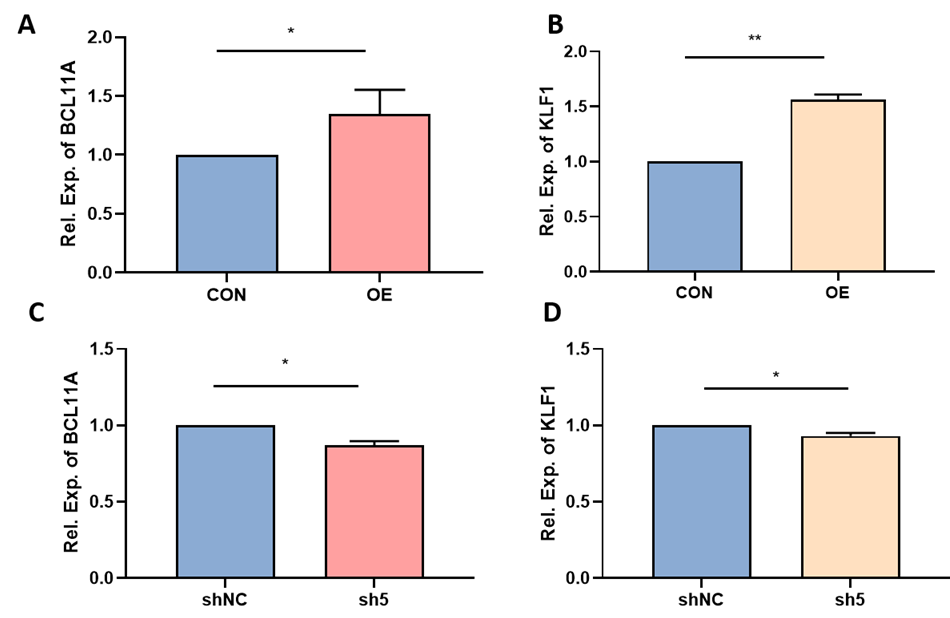


**Supplemental Figure 2: Effects of *ANTXR1* overexpression or knockdown on *BCL11A* and *KLF1*** **mRNA levels in K562 cells.** (**A, B**) *ANTXR1* was overexpressed in K562 cells using a pHAGE-fEF-1a-IRES-ZsGreen-2 vector. Cells transfected with an empty vector served as controls. The effects of *ANTXR1* overexpression on *BCL11A* and *KLF1* expression measured by RT-PCR. Overexpression of *ANTXR1* in K562 cells, significantly increased *BCL11A* and *KLF1* expression compared to that observed in the control group; (**C, D**) ANTXR1-sh5 or NC-shRNA vector transfected K562 cells. The effects of *ANTXR1* knockdown on *BCL11A* and *KLF1* expression measured by RT-qPCR. Knockdown of *ANTXR1* in K562 cells significantly decreased the expression of *BCL11A* and *KLF1* compared with that observed in the control group. Three independent assays were performed with the error bar representing the SD. **P*<0.05，***P*<0.01.
